# Association between animal welfare indicators and mastitis pathogens in bulk tank milk: the reality of small- and medium-scale dairy cattle farms in Italy

**DOI:** 10.3389/fvets.2026.1860743

**Published:** 2026-08-20

**Authors:** Dáša Schleicherová, Jatziri Mota-Gutierrez, Claudio Forte, Laura Ozella, Francesca Fusi, Mario Vevey, Stefania Bergagna

**Affiliations:** 1 Istituto Zooprofilattico Sperimentale del Piemonte, Liguria e Valle D’Aosta, Turin, Italy; 2 Dipartimento di Scienze Veterinarie, Università di Torino, Grugliasco, Turin, Italy; 3 Istituto Zooprofilattico Sperimentale della Lombardia e dell’Emilia Romagna “Bruno Ubertini” (IZSLER), Brescia, Italy; 4 Associazione Nazionale Bovini di Razza Valdostana, Gressan, Italy

**Keywords:** animal welfare, cattle management practice, ClassyFarm, herd size, microbial community, welfare quality protocol for cattle

## Abstract

**Introduction:**

Animal welfare is increasingly recognized as a key determinant of dairy herd health, yet its relationship with the presence of mastitis pathogens at the herd level remains insufficiently explored. This study aimed to evaluate the association between animal welfare indicators and the presence of major mastitis pathogens in bulk tank milk (BTM) from dairy cattle herds in the Aosta Valley, Italy, while also characterizing microbial profiles using bacteriological culture and polymerase chain reaction (PCR).

**Methods:**

A broad microbiological screening was performed on unique BTM samples from 490 individual farms. Concurrently, a subset of 355 small- and medium-scale dairy cattle farms underwent detailed animal welfare assessments using the standardized ClassyFarm protocol. Data were collected by experienced assessors in northern Italy in 2022, with each farm visited once.

**Results:**

The median lactating herd size was 33 cows (IQR: 57–18) and included Aosta Red Pied and Aosta Black Pied cattle breeds. All farms were tie-stall in the Aosta Valley, where herds are grazed outdoors for most of the year and kept indoors in tied-stalls during winter. BC identified at least one pathogen in 73.5% of samples, with most positive samples exhibiting low diversity, predominantly single (48.6%) or dual-species (41.1%) profiles. *Staphylococcus aureus* (*N* = 281) and *Streptococcus agalactiae* (*N* = 148) were the most frequently isolated pathogens. In contrast, PCR revealed prevalence of polymicrobial contamination, with the majority of samples harboring four or more bacterial species and 44.4% containing ≥5 species. Logistic regression models built using the binary PCR outcomes for the 355-farm subset demonstrated consistent negative associations between animal welfare indicators and the presence of both *S. agalactiae* and *S. aureus*. Higher total animal welfare scores were associated with reduced odds of detection (*S. agalactiae*: OR = 0.943, *p* ≤ 0.001; *S. aureus*: OR = 0.948, *p* = 0.005), as were animal-based measures, farm management, and housing and equipment scores.

**Discussion:**

These findings provide evidence that improved animal welfare is associated with a lower likelihood of major mastitis pathogens in BTM.

## 1 Introduction

High levels of animal welfare in dairy farming are widely recognized as a cornerstone of sustainable livestock production, with well-documented benefits for animal health, productivity, milk quality, and overall farm efficiency (1). Cows kept under adequate welfare conditions generally experience reduced chronic stress, improved immune competence, and greater resilience to environmental and managerial challenges, ultimately contributing to the improved performance and economic sustainability of dairy systems. Despite these advantages, ensuring high welfare standards remains challenging due to the complexity of modern dairy management, which includes housing design, feeding strategies, hygiene practices, disease prevention, and continuous monitoring of behavioral and physiological indicators. These challenges are particularly evident in small- and medium-scale dairy farms, where structural and economic constraints frequently limit the adoption of advanced welfare technologies and infrastructure upgrades (2), although this may sometimes be offset by more intensive individual animal supervision by the stockperson. Given this variability in management and infrastructure, there is a critical need for standardized monitoring to ensure consistent welfare outcomes.

In Europe, several structured assessment tools have been developed to objectively evaluate farm-level welfare conditions. Among these, the ClassyFarm system represents a comprehensive framework integrating resource-based indicators, such as housing characteristics, equipment quality, and management procedures, with animal-based measures reflecting the animals’ physical and behavioral condition, including cleanliness, injuries, lameness, health status, and social behavior (3). By combining these components across three specific assessment areas, ClassyFarm generates an overall welfare score that classifies farms into defined risk categories, supporting both regulatory oversight and farm-level decision-making. Recent Italian studies highlight the importance of standardized animal welfare assessment tools for identifying management- and welfare-related risks in cattle production systems (3–6).

The ClassyFarm system, developed by the Italian Ministry of Health, integrates resource-based indicators and animal-based measures within a structured risk classification framework applicable across different housing systems and herd sizes (3). In small- and medium-scale dairy farms typical of the Aosta Valley, ClassyFarm accurately captures variability related to farm management, staff training, housing conditions, and animal-based outcomes, with most farms classified as adequate or optimal despite structural constraints of mountain environments (3). Expert evaluations have shown that management-related factors, such as group size and homogeneity, access to resources, frequency of daily inspections, hygiene and handling practices, exert a greater impact on cattle welfare than housing characteristics alone (6). Animal-based measures, including lameness, body condition score, and mortality rate, remain among the most informative indicators of severe welfare impairment. These findings support the use of integrated welfare assessment protocols like ClassyFarm, which prioritize management quality and key animal-based indicators, particularly in small and heterogeneous herds. The effectiveness of these protocols is most evident when monitoring high-impact clinical conditions.

Among health conditions affecting dairy cow welfare, mastitis remains one of the most significant and economically costly diseases worldwide. Its impact is considerable, due to reduced milk yield, impaired milk quality, increased treatment costs, and higher culling rates, while also causing pain, inflammation, and behavioral alterations that directly compromise animal well-being (7). Globally, mastitis epidemiology reflects a complex interplay between host, environment, and pathogen management, with average herd-level prevalence rates for subclinical mastitis frequently ranging from 20% to over 50% in multi-regional surveys. Mastitis prevalence and severity are strongly influenced by housing hygiene, management practices, and milking routines, underscoring the close link between farm-level welfare and udder health. Recent studies consistently identify *Staphylococcus aureus* and *Streptococcus agalactiae* as two of the most prevalent pathogens responsible for subclinical mastitis in dairy herds in Europe, North America, Asia, and Africa (8–10).

In Italy, these contagious pathogens present persistent challenges, particularly within northern regional production systems. For instance, in neighboring Lombardy, monitoring initiatives have historically highlighted herd-level *S. agalactiae* prevalence rates between 7.3% and 17.2% (11). While previous veterinary research in Italy has extensively utilized the national ClassyFarm protocol to map animal welfare indicators against somatic cell counts or clinical outbreaks, these investigations have been overwhelmingly concentrated in the intensive, large-scale, loose-housing dairy operations of the Po Valley plain. Consequently, there is a profound scarcity of data evaluating how these institutionalized welfare indicators correlate with pathogen pressure inside traditional small- and medium-scale Alpine dairy systems, such as those found in the Aosta Valley. Within these specific mountain frameworks, managing contagious pathogens is uniquely complicated by regional herd structures, traditional tie-stall housing constraints, and seasonal pasture movements (12).

Recent molecular evidence in Italy also indicates that *S. agalactiae* can adapt to environmental and fecal–oral transmission pathways rather than spreading solely via contagious milking contact (11). This highlights a critical intersection where farm-level welfare scores, such as those evaluating housing and hygiene, directly influence pathogen pressure. Meta-analytical evidence indicates that the prevalence of *S. aureus*, including methicillin-resistant strains, varies across regions but is consistently associated with long-term herd conditions rather than sporadic clinical cases (13). In addition, recent studies on microbial communities in dairy environments have shown close associations between pathogens detected in milk and environmental hygiene and welfare-related factors, supporting the use of microbiological indicators as complementary tools in welfare monitoring programs (2). The increasing occurrence of antimicrobial-resistant strains of both *S. aureus* and *S. agalactiae*, along with their ability to form biofilms and persist within mammary tissues, has further heightened concerns for animal and public health in European production systems. Consequently, this reinforces the importance of preventive strategies based on management and welfare improvements rather than therapeutic interventions alone (8, 9).

Despite the growing body of literature on dairy cow welfare and mastitis epidemiology, empirical evidence directly linking standardized welfare scores, farm characteristics, and objective microbiological outcomes remains limited. This gap is particularly evident in small- and medium-scale dairy systems, which are often underrepresented in large epidemiological studies but represent a substantial proportion of dairy farms in several European regions. While definitions vary, small-scale dairy farms are generally characterized by traditional housing and smaller herd sizes (often <50 cows) (14), yet, they are essential for the production of traditional dairy products. In mountain and marginal areas, such as the Aosta Valley, dairy production is typically characterized by small- and medium-scale herd sizes, heterogeneous management practices, and variable access to resources. Recent studies suggest that welfare outcomes, milk quality, and farm performance in small-scale mountain systems are strongly influenced by biosecurity and management practices, rather than by herd size alone (15, 16). Integrating animal welfare assessment with microbiological surveillance may therefore provide a more comprehensive understanding of herd health dynamics and management effectiveness. However, empirical data supporting this integrated approach remain scarce, particularly for small- and medium-scale dairy farms.

To address this gap, the present study utilized bulk tank milk (BTM) samples collected from each farm and analyzed them using both bacterial culture and real-time PCR to detect the presence of common mastitis pathogens. PCR offers the advantage of being faster and more sensitive, providing results within hours and allowing for the simultaneous detection of multiple pathogens in a single sample. However, it detects genetic material, including non-viable bacteria, which could sometimes result in false positives. On the other hand, bacteriological culture, while slower (requiring up to 48 h for results), is the gold standard for confirming the presence of viable pathogens. Culture also provides additional information, such as the antibiotic resistance of the pathogens, which is crucial for determining appropriate treatment (17). However, the sensitivity of culture can be limited when bacterial loads are low or in mixed infections. By combining PCR and bacteriological culture, this study benefits from the comprehensive detection provided by PCR while ensuring reliable confirmation of pathogen viability through traditional culture methods. Overall, a recent study on small-scale mountain dairy farms in the Aosta Valley suggested that animal welfare outcomes are associated with management quality and farmers’ engagement and satisfaction, rather than structural farm characteristics (18). However, that prior research focused strictly on the drivers of welfare scores, leaving a critical knowledge gap regarding whether these management-driven welfare outcomes translate into tangible differences in infectious disease pressure. Integrating standardized welfare scores with objective microbiological outcomes is highly needed to validate these systems, yet data directly linking welfare metrics to specific pathogen prevalence remain scarce.

The present study couples comprehensive welfare indicators with objective, PCR-detected pathogen profiles across 355 dairy farms, moving beyond behavioral indices to quantify the direct protective effects of welfare on herd infection dynamics. Specifically, we aimed to: (i) assess animal welfare scores across 355 dairy farms located in the Aosta Valley, Italy using the standardized ClassyFarm system; (ii) estimate the prevalence of mastitis-causing pathogens, with a specific focus on *S. aureus* and *S. agalactiae* in BTM from the same farms; and (iii) examine the relationship between animal welfare scores and specific pathogen presence with the aim of identifying potential biological indicators of herd welfare and providing evidence-based insights for improving management strategies in small- and medium-scale dairy systems.

## 2 Materials and methods

The study was conducted in two distinct phases, utilizing a single BTM sample *per* farm. In the first phase, a broad microbiological screening was performed on unique BTM samples from 490 dairy farms to evaluate pathogen detection results obtained by bacteriological culture and Real-Time PCR. These 490 farms represent the population participating in routine regional milk quality monitoring. In the second phase, a subset of 355 farms accepted to examine the relationship between lactating herd welfare and pathogen presence. This subset represented farms for which comprehensive, official animal welfare data via the ClassyFarm protocol were available. Crucially, the binary outcomes (presence/absence) generated by the highly sensitive Real-Time PCR assay were utilized as the primary microbiological target in the regression models. The remaining 135 screened farms were excluded from the association models due to a lack of concurrent welfare records. The study population was restricted to dairy production sectors. For each participating farm, herd size was defined strictly as the number of lactating dairy cows actively contributing to the BTM at the time of sampling. Animals dedicated to meat production, dry cows, and young replacement stock were excluded from this denominator, as they do not contribute to the BTM volume or its microbiological profile.

### 2.1 Milk sample

BTM samples were collected upon delivery to 490 dairy cattle herds located in the Aosta Valley Region, Italy using sterile disposable tubes. After collection, all samples were immediately cooled (+2 °C to +8 °C) and transported within 24 h to the Laboratory of Animal Welfare, Istituto Zooprofilattico Sperimentale del Piemonte, Liguria, Valle d’Aosta. In cases where it was not possible to deliver the samples to the laboratory within 24 h, samples were frozen and kept at −20 °C. Once they arrived at the laboratory, refrigerated samples were analyzed, preferably within 48 h, while frozen samples were thawed prior to analysis. While storage at −20 °C is known to potentially reduce cell viability for traditional bacteriological culture, it effectively preserves bacterial DNA for molecular detection. Because the downstream GLM regression models mapping welfare indicators to pathogen presence relied exclusively on real-time PCR outcomes rather than live culture isolation, this storage protocol maintained high diagnostic consistency and prevented systematic bias in the statistical analyses.

BTM was used as a proxy for herd-level pathogen prevalence, providing a representative measure of microbial exposure across the entire herd (13). This approach allows efficient assessment of the herd’s microbiological status without the need for individual sampling of all cows, and reflects the overall management, hygiene, and health conditions affecting the herd.

### 2.2 Bacterial culture

For the qualitative bacteriological examination, 100 μL of each milk sample was spread on Columbia Blood Agar, Baird Parker agar, Tallium Kristalviolette Toxin agar, Prototheca Isolation Medium, McConkey agar, and Gassner agar. The agar plates were incubated aerobically at 37 ± 2 °C. The cultures were examined after 18–24 h and 42–48 h, respectively. Bacterial identification followed a structured, sequential diagnostic workflow. Initial screening was based on colony morphology, hemolytic patterns on blood agar, Gram staining, and catalase testing. Following initial categorization, specific confirmation assays were deployed: the CAMP test was used exclusively to confirm presumptive *S. agalactiae* isolates; serum agglutination tests were utilized for the serological typing and confirmation of *S. aureus* and specific streptococcal groups; and Matrix-Assisted Laser Desorption Ionization–Time of Flight Mass Spectrometry (MALDI-TOF MS) was applied as the definitive identification tool for *S. aureus*, atypical colonies, *Mycoplasma* species, and environmental isolates requiring high-resolution proteomic profiling. The comprehensive mapping of each diagnostic test to its respective target microorganism is detailed in Supplementary Table 1. All procedures followed standardized microbiological protocols to ensure accuracy and reproducibility of results.

### 2.3 Real-time PCR

A commercial real-time PCR test kit (PathoProof Mastitis PCR Assay, Thermo Fisher Scientific) was used for direct analysis of all BTM samples, without any prior bacterial culture steps. A total of 200 μL of milk was used as starting material for DNA extraction, following the manufacturer’s instructions. The extraction procedure included enzymatic lysis to disrupt the cell walls of both gram-positive and gram-negative bacteria, followed by spin column–based DNA purification and elution. The assay comprised four separate multiplex Real-Time PCR reactions, targeting 11 bacterial species and groups, covering more than 25 mastitis-causing species in total:

- **Reaction 1:** *S. aureus*, *Corynebacterium bovis*, *Enterococcus* spp. (including *Enterococcus faecalis* and *Enterococcus faecium*), and *Mycoplasma bovis.*
- **Reaction 2:** *Streptococcus dysgalactiae*, *Escherichia coli*, and *Mycoplasma* spp.
- **Reaction 3:** *Prototheca* spp., *Streptococcus uberis*, *S. agalactiae*, and *Staphylococcus* spp. [including non-aureus staphylococci (NAS)].
- **Reaction 4:** *Klebsiella* spp. (including *K. pneumoniae* and *K. oxytoca*), *Arcanobacterium pyogenes*/*Peptoniphilus indolicus*, *Serratia marcescens*, and yeast (17).

The assay allowed identification of bacteria to the species or group level; for example, all NAS were detected in a single PCR reaction and instrument channel, while *A. pyogenes* and *P. indolicus* were also detected together in one reaction. Internal amplification controls (IAC) were included to confirm acceptable PCR reaction conditions, and a negative control (distilled H_2_O) was run with every batch to monitor potential cross-contamination. Real-Time PCR was selected for its high sensitivity and specificity, enabling rapid and accurate detection of mastitis-causing pathogens at the herd level. The outcomes of this detection method were associated with animal welfare assessment.

### 2.4 Animal welfare assessment

Animal welfare assessments were conducted following the instructions of the ClassyFarm checklist (15). Minor modifications were made for practical or statistical reasons (e.g., non-lactating cows were excluded). Farm visits usually started at 08:00 h (±1 h) after morning milking and lasted, depending on herd size, 6 to 13 h. Data collection was performed at each farm in a fixed order.

The total welfare checklist was composed of resource- and animal-based indicators, listed in a multiple-choice checklist including 75 items divided into three areas of assessment:

- **Area A:** farm management and staff training (25 items)
- **Area B:** housing and equipment (28 items)
- **Area C:** animal-based measures (22 items)

Thirty-eight items out of 75 are based on minimum requirements established by current national and European legislation (19, 20). Each indicator has two (insufficient and acceptable) or three assessments (insufficient, acceptable, and optimal). Thresholds between the different levels of judgment are based on the possibility for animals to meet their biological needs and enjoy the five freedoms (1, 21). Each indicator level has a different weight according to its potential impact on dairy cow welfare (1). Based on the assessment performed on-farm, area (or section) scores were calculated by summing the scores of the indicators belonging to each area. The total welfare score was then calculated considering a 50% contribution from Area A and Area B, and the remaining 50% from Area C. The total welfare and subsection scores are expressed in percentages from 0 to 100, representing the farm’s risk level (1). Recent systematic reviews on welfare indicators also discuss the validity and relative importance of different measures in assessment protocols (22). A cross-sectional design was adopted to provide a snapshot of herd welfare across all farms, allowing evaluation of potential associations between management practices, animal-based measures, and microbial outcomes in a practical and time-efficient manner (23). Although ClassyFarm provides a standardized and comprehensive assessment of dairy cow welfare, some indicators rely on observational scoring, which may introduce variability. Nevertheless, it represents the most validated and widely used system for on-farm welfare assessment in Italy, providing consistent and comparable results across diverse farms (1).

### 2.5 Statistical analysis

Statistical analysis was performed using R software (version 3.3.2), considering an alpha level of 5% (29). To demonstrate the statistical validity and power of our cross-sectional design, a post-hoc sample size justification was calculated using Cochran’s formula adjusted for a finite population. Given the unique, enclosed geography of the Aosta Valley, the regional target population of active dairy and mixed-production holdings is finite (*N* = 650). Assuming a conservative expected pathogen prevalence of 50% (which maximizes the sample size requirement), a 95% confidence level (*Z* = 1.96), and a standard 5% margin of error, the minimum required sample size for robust inferential analysis was determined to be 241 farms. Our analytical dataset of 355 farms vastly exceeds this threshold, capturing more than half of the region’s active production units. This elevated sample density effectively compresses our actual margin of error to 3.5%, ensuring excellent statistical power for the downstream generalized linear models (GLM) and eliminating the risk of type II statistical errors.

A chi-square test was used to evaluate the distribution of positive and negative results within each diagnostic method (bacteriological culture and PCR) at farm level. Each BTM sample was classified as positive or negative according to each method separately. Univariable logistic regression models were used to assess the association between microbial presence (binary outcome: 0 = absent, 1 = presence generated by Real-Time PCR) and each farm welfare-related variable: Animal welfare score, Animal-based measures, Farm Management, and Housing and Equipment. Each variable was analyzed separately using a generalized linear model (GLM) with binomial distribution and a logit link function using the glm() function in R (24, 25). The log-odds of microbial presence per 1% increase in the predictor were estimated. Coefficients were exponentiated to obtain odds ratios (ORs) for interpretability. 95% confidence intervals for ORs were calculated from the standard errors of the regression coefficients. Statistical significance was set at *p* < 0.05. To account for potential confounding due to differences in lactating herd demographic structure, lactating herd structure was accounted for using proportional indicators (proportion of lactating cows, dry cows, and calves). Lactating herd size was categorized into three classes (<50, 51–100, and 101–162 cows) to allow assessment of potential non-linear effects and effect modification in a biologically interpretable framework. These cut-offs were chosen based on the distribution of lactating herd sizes in the dataset, ensuring adequate sample size within each stratum while reflecting meaningful differences in herd management scale commonly used in herd health epidemiological studies. This formulation was used to avoid multicollinearity arising from linear dependency among raw herd counts. Variance inflation factors were used to assess collinearity, confirming no relevant multicollinearity among predictors (VIF < 2 for all variables). Interaction terms between lactating herd size categories and welfare indicators were included in separate GLM models. This combination of methods allows a robust assessment of the relationships between management practices, welfare indicators, and microbial outcomes at the lactating herd level.

## 3 Results

The study population consisted of 355 small- and medium-scale dairy farms that completed both microbiological and animal welfare assessments. The mean lactating herd size was 42 animals, whereas the median was 33 cows (IQR: 18–57). The demographic structure of these lactating herds was characterized by a milking cohort averaging 13 lactating dairy cows and 12 dry cows, alongside 11 heifers and 6 calves. The lactating herd population was classified into three size categories (C1–C3) based on the number of animals *per* farm, with a relatively balanced distribution across groups (C1: 127 farms; C2: 116 farms; C3: 112 farms). The production systems were nearly evenly split between specialized and diversified systems, with 183 farms being classified as milk-only production systems and 172 farms operating as mixed-production systems. Productivity across the sampled farms reflected a low-intensity management style, with a mean daily milk yield of 15.47 ± 25.38 kg/day *per* cow. These descriptive parameters reinforce the classification of the sampled farms as traditional, small- and medium-scale holdings typical of the regional agricultural landscape.

### 3.1 Phenotypic profile via bacteriological culture of bulk tank milk

Of the 490 milk samples analyzed, bacteriological culture identified 360 positive samples, 94 negative samples, and 36 with polymicrobial contamination. Among culture-positive samples, most contained a single bacterial species (175/360), followed by two species (148/360), with fewer samples showing three or more species (31 and 6/360, respectively). *S. aureus* was the most frequently isolated pathogen, appearing in 281 total samples, predominantly in pure or dual-culture states, followed by *S. agalactiae* (*n* = 148) and NAS (*n* = 48, Table 1). Statistical analysis revealed no significant differences between farms regarding the overall prevalence of positive samples detected using bacteriological culture (360 vs. 490, *χ*^2^ = 42.15, *p* = 0.108).

**Table 1:** Number of bulk tank milk samples positive for each pathogen by bacteriological culture and culture purity in 490 Italian dairy cattle.

Pathogen	Pure	2-species	3-species	4-species	≥5-species	Total
*Staphylococcus aureus*	123	128	24	4	2	281
*Streptococcus agalactiae*	17	100	25	4	2	148
*Streptococcus dysgalactiae*	3	12	4	2		21
*Streptococcus uberis*	6	15	7	1	2	31
Non-aureus staphylococci	23	16	7	2		48
*Mucorales*	2	18	18	2	2	42
*Klebsiella*	1	4	2	1		8
*Serratia*		1	1			2
*Streptococcus Group D*		1				1
*Escherichia coli*		2	3	1	1	7
*Enterococcus* spp.			1			1
*Enterobacter*			1		1	2

### 3.2 Occurrence of opportunistic pathogens in bulk tank milk from dairy cattle herds detected using real-time PCR

Of the 490 milk samples analyzed, Real-Time PCR identified 483 positive samples and 7 negative samples. Among culture-positive samples, most contained more than five species (214/483), followed by four species (104/483), with fewer samples showing three, two or single species (87, 50 and 28/483, respectively). Among individual pathogens, NAS were the most frequently detected (391 positives), followed by *S. aureus* and *Enterococcus* spp. (Table 2). Streptococcal species were also prevalent, with *S. uberis* (249), *S. agalactiae*, and *S. dysgalactiae* detected across multiple samples (Table 2). Other bacteria, including *Corynebacterium* spp., *Trueperella* spp., yeasts, *Klebsiella* spp., *Serratia* spp., *Mycoplasma* spp., and *Prototheca* spp., were less common and typically appeared in samples with four or more species. Statistical analysis revealed no significant differences between farms regarding the overall prevalence of positive samples detected using Real-Time PCR (483 vs. 490, *χ*^2^ = 12.84, *p* = 0.998).

**Table 2:** Number of bulk tank milk samples positive for each pathogen by Real-Time PCR and number of species per sample in 490 Italian dairy cattle.

Pathogen	Pure	2-species	3-species	4-species	≥5- species	Total
*Enterococcus* spp.	9	24	61	82	191	367
*Staphylococcus aureus*	5	25	60	86	204	380
*Streptococcus dysgalactiae*	1	4	11	25	121	162
*Escherichia coli*	2	4	5	10	100	121
*Streptococcus agalactiae*	1	5	18	47	139	210
*Streptococcus uberis*	5	8	20	52	164	249
Non-aureus staphylococci coagulase	4	26	66	85	210	391
*Serratia*	1	0	0	1	5	7
*Corynebacterium bovis*	0	3	16	16	71	106
*Mycoplasma*	0	0	0	0	5	5
*Prototheca*	0	0	0	0	2	2
*Trueperella*	0	0	2	5	29	36
Yeasts	0	0	3	4	28	35
*Klebsiella*	0	0	1	1	7	9
Total	28	99	263	414	1,276	2080

### 3.3 Relationship between measures of animal welfare total scores of dairy cattle farms and bacterial occurrence in bulk tank milk detected using PCR-based methods

The ClassyFarm assessment yielded a mean total welfare score of 76.95 ± 6.85% from 355 farms. Regarding the distribution of welfare scores, 70.99 ± 10.74% of the farms were classified as Adequate ranging scores from 61% to 80%, 28.17 ± 11.38% as Optimal ranging scores from 81% to 100%. Analysis of the specific welfare areas showed that Animal-Based Measures achieved the highest mean score (84.72 ± 9.20%), followed by facilities and equipment and Personal and corporate management (72.03 ± 8.95 and 66.53 ± 8.46%, respectively), while the lowest performance was observed in the Great Risks area (52.56 ± 13.39%). Among the 355 farms included in the regression analyses, PCR detected *S. aureus* in 273 farms and *S. agalactiae* in 152 farms. Overall, the GLM results showed that higher welfare scores were associated with a modest reduction in the odds of bacterial presence, but statistically significant effects were observed only for *S. aureus* and *Streptococcus agalactiae* (Tables 3, 4).

**Table 3:** Logistic regression results showing the association between animal welfare indicators and *Staphylococcus aureus* presence in bulk milk samples from 355 Italian dairy cattle.

Predictor	Beta	SE	*Z*-value	*p*-value	OR	CI_lower	CI_upper
Overall Animal Welfare	−0.052	0.019	−2.760	0.005	0.948	0.913	0.984
Animal-based measures	−0.030	0.014	−2.093	0.036	0.969	0.942	0.998
Farm management	−0.033	0.014	−2.311	0.020	0.966	0.939	0.994
Housing and equipment	−0.028	0.014	−1.977	0.048	0.971	0.944	0.999

Data refers to the subset of 355 farms with complete ClassyFarm animal welfare assessments. Real-Time PCR detection results are reported at farm level. Beta: estimated regression coefficient, indicating the direction and magnitude of association. SE: standard error of the Beta estimate. Z-value: test statistics for the null hypothesis that Beta = 0. *p*-value: significance level of the association. OR: odds ratio, representing the change in odds of the outcome per unit increase in the predictor. CI_lower/CI_upper: lower and upper bounds of the 95% confidence interval for the odds ratio. Lactating herd composition variables were not significantly associated with the outcome.

Interpretation: OR < 1 indicates a negative association with the outcome, OR > 1 indicates a positive association, and statistically significant predictors are highlighted by *p* < 0.05.

**Table 4:** Logistic regression results showing the association between animal welfare indicators and *Streptococcus agalactiae* presence in bulk milk samples from 355 Italian dairy cattle.

Predictor	Beta	SE	*Z*-value	*p*-value	OR	CI_lower	CI_upper
Overall Animal Welfare	−0.058	0.016	−3.511	< 0.001	0.943	0.912	0.974
Animal-based measures	−0.029	0.011	−2.448	0.014	0.971	0.949	0.994
Farm Management	−0.048	0.013	−3.498	< 0.001	0.952	0.927	0.978
Housing and equipment	−0.030	0.012	−2.454	0.014	0.970	0.947	0.993

Data refers to the subset of 355 farms with complete ClassyFarm animal welfare assessments. Real-Time PCR detection results are reported at farm level. Abbreviations. Beta: estimated regression coefficient, indicating the direction and magnitude of association. SE: standard error of the Beta estimate. *Z*-value: test statistics for the null hypothesis that Beta = 0. *p*-value: significance level of the association. OR: odds ratio, representing the change in odds of the outcome per unit increase in the predictor. CI_lower/CI_upper: lower and upper bounds of the 95% confidence interval for the odds ratio. Lactating herd composition variables were not significantly associated with the outcome.

Interpretation: OR < 1 indicates a negative association with the outcome, OR > 1 indicates a positive association, and statistically significant predictors are highlighted by *p* < 0.05.

In detail, for *S. aureus*, higher overall welfare was significantly associated with reduced odds of *S. aureus* presence (*p* = 0.005), corresponding to approximately a 5% reduction in odds for each 1% increase in animal welfare scores. Similarly, higher scores in animal-based measures (*p* = 0.036) were associated with a ~ 3% reduction in odds per 1% increase. Farm management (*p* = 0.021), and housing and equipment (*p* = 0.048) also showed protective effects, each corresponding to a ~ 3% reduction in odds per 1% increase. For *S. agalactiae*, higher overall welfare scores were significantly associated with reduced odds of this bacterium (*p* ≤ 0.001), representing a ~ 6% reduction in odds per 1% increase. Additional significant predictors included animal-based measures (*p* = 0.014) corresponded to ~3% reduction per 1% increase, while farm management (*p* ≤ 0.001), and housing and equipment (*p* = 0.014) were associated with ~3–5% reductions per 1% increase. No significant interactions between lactating herd size and welfare indicators were observed (all *p* > 0.190), indicating that associations with mastitis pathogens were consistent across lactating herd size categories (data not shown).

For *Enterococcus* spp., *Corynebacterium bovis*, *S. dysgalactiae*, *Mycoplasma* spp., *E. coli*, *S. uberis*, *Staphylococcus* spp., *Serratia marcescens*, and *Klebsiella* spp., detected using PCR-based methods none of the welfare predictors reached statistical significance (*p* > 0.05, data not shown), although most ORs were below 1.

## 4 Discussion

The present study provides evidence of a significant association between animal welfare scores and the presence of *S. aureus* and *S. agalactiae* in BTM detected using PCR from dairy farms in the Aosta Valley. Lower welfare scores were consistently associated with higher odds of pathogen detection, highlighting the role of management quality, housing conditions, and hygiene practices in influencing both animal health and microbiological outcomes (7, 9, 26). It is worth noting that the individual odds ratios for the welfare indicators hover between 0.94 and 0.97. While these effect sizes appear close to unity on a *per*-unit basis, their practical significance becomes clearer when evaluated across broader shifts on the welfare score scale. For instance, a 1% incremental increase in housing scores yields a modest 3% reduction in the odds of *S. aureus*. However, a realistic management intervention that improves a farm’s housing score by 10 percentage points would result in a cumulative, biologically meaningful reduction in the odds of pathogen presence (approx. 26% reduction). Therefore, while the immediate impact of minor welfare shifts is small, sustained systemic improvements across welfare categories offer a viable, non-antibiotic pathway toward reducing mastitis pathogen pressure on dairy farms. Unlike other mastitis-associated microorganisms, *S. aureus* and *S. agalactiae* are strongly linked to long-term factors such as milking routines, housing conditions, and preventive health strategies, making their detection in BTM a meaningful indicator of cumulative herd-level management and welfare (7–9, 27).

Improved welfare conditions likely reduce pathogen transmission through multiple mechanisms, including enhanced milking hygiene, maintenance of teat integrity, and reduced stress-induced immunosuppression, all of which can influence susceptibility to *S. aureus* and *S. agalactiae* infection (8–10). The strong association observed for *S. aureus*, compared to other pathogens such as *E. coli* or *S. uberis*, likely reflects its contagious nature and dependence on herd management practices, whereas environmental pathogens are more influenced by external conditions. It is noteworthy that when interpreting these findings, it is critical to account for the inherent characteristics of the diagnostic modalities employed. While the real-time PCR assay offered exceptional sensitivity and a robust window for detecting herd-level exposure to *S. aureus* and *S. agalactiae*, its target is microbial DNA rather than viable bacterial cells. Consequently, the inferential associations modeled in this study reflect the presence of specific genetic material in the BTM, not necessarily active, viable infections within the mammary tissues at the exact moment of sampling. DNA fragments can persist in milk following successful therapeutic clearance or enter the bulk tank via non-viable environmental contaminants. Therefore, while our models demonstrate a highly significant link between superior farm welfare scores and reduced pathogen DNA pressure, these results should be interpreted with caution and should not be directly extrapolated to imply a definitive reduction in live, culture-isolated clinical mastitis rates.

Lactating herd size was not significantly associated with either welfare scores or microbial occurrence detected using PCR. Furthermore, interaction analyses showed that lactating herd size and composition did not modify the relationship between welfare indicators and pathogen detection, indicating that the protective effects of higher welfare scores were consistent across small- and medium-sized farms. This finding suggests that farm scale alone is not a reliable proxy for animal welfare or udder health, and that smaller farms can achieve high standards when appropriate management practices are implemented (28). This is particularly relevant for traditional Italian dairy farms, where management variability is high and targeted interventions can produce meaningful improvements without structural expansion. The results of the generalized linear model further support the robustness of this association, showing that all major components of the welfare assessment, animal-based measures, farm management, and housing and equipment, were significantly and negatively associated with *S. aureus* and *S. agalactiae* presence. These findings suggest that achieving higher welfare standards may act as a protective factor against contagious mastitis, reinforcing the value of a multidimensional approach to lactating herd health assessment. The higher prevalence of polymicrobial contamination observed by PCR further emphasizes that welfare interventions may impact overall microbial dynamics in BTM, not just individual pathogens. This observation highlights the importance of integrating multiple assessment methods to comprehensively understand herd-level pathogen dynamics.

Despite these strengths, several limitations should be considered. First, the use of BTM sampling does not allow identification of infected individual animals; however, it is widely recognized as a practical and cost-effective proxy for herd-level udder health and pathogen circulation (27). Each farm was represented by a single BTM sample, providing only a cross-sectional snapshot of pathogen presence and limiting the ability to capture temporal variability within farms. In addition, the comparison of detection frequencies between bacteriological culture and PCR was descriptive in nature and based on single-time-point classifications; therefore, it does not allow formal assessment of diagnostic performance or agreement between methods. Finally, the relatively low number of repeated observations *per* farm restricts the possibility of evaluating within-farm consistency of pathogen detection over time.

Additionally, the classification of lactating herd size was based on Italian production systems; therefore, caution is warranted when extrapolating these findings to other countries with different structural characteristics (21). Breed-related effects were not considered a confounding factor, as all farms included in the study raised Aosta Red Pied (Valdostana Pezzata Rossa) and Aosta Black Pied (Valdostana Pezzata Nera) cattle. Finally, due to the observational design, causal relationships cannot be established, and the associations observed should be interpreted with caution. Although the present study focused on aggregated ClassyFarm indicators to ensure model stability and reduce issues related to multiple testing and collinearity among individual items, a more detailed analysis at the level of single items within each assessment area could provide additional biological insight into specific welfare–disease pathways. Therefore, future studies should explore indicator-level associations within ClassyFarm domains to refine understanding of specific risk factors for mastitis pathogens and to enhance the generalizability of findings to countries using different welfare assessment frameworks.

A key strength of this study is the integration of standardized welfare assessment with microbiological data at the lactating herd level across a large number of commercial farms, providing both practical and research-relevant insights. Overall, the combination of pathogen detection in BTM detected using PCR with welfare scores offers a multidimensional perspective for lactating herd health monitoring. These findings suggest that improving welfare conditions serves as a practical strategy to reduce the prevalence of contagious mastitis, particularly in small- and medium-scale farms where management practices may vary despite similar herd sizes (7, 9, 26). Integrating biological indicators with welfare assessment thus provides a valuable tool for proactive herd health management.

## 5 Conclusion

Animal welfare scores were found to be significantly associated with the presence of *S. aureus* and *S.agalactiae* in BTM, highlighting the success of management practices in promoting udder health and overall animal welfare. These results confirm that combining animal welfare assessments with microbiological indicators, such as *S. aureus* and *S. agalactiae* occurrence, provides a practical and biologically meaningful tool for monitoring lactating herd health and guiding evidence-based management strategies, particularly in small- and medium-scale dairy systems. Future studies should investigate additional mastitis-causing pathogens, include larger and more diverse herd sizes, and adopt longitudinal designs to clarify causal relationships between animal welfare, management practices, and microbial dynamics, supporting sustainable interventions to improve both animal health and productivity.

## Data Availability

The data used in this study belong to the Istituto Zooprofilattico Sperimentale del Piemonte, Liguria e Valle D’Aosta and Associazione Nazionale Bovini di Razza Valdostana. The dataset contains sensitive information related to animal health surveillance and is subject to institutional confidentiality and legal restrictions. Consequently, the authors are not authorized to share the dataset publicly. Requests for data access may be directed to the Institute's administration at stefania.bergagna@izsplv.it, subject to their internal evaluation and data use policies.
