# Supplementary material for: Association between animal welfare indicators and mastitis pathogens in bulk tank milk: the reality of small- and medium-scale dairy cattle farms in Italy
Source: Front Vet Sci. 2026 Aug 20;13:1860743. doi: 10.3389/fvets.2026.1860743 (PMC13538020; doi:10.3389/fvets.2026.1860743)
Supplement: Supplementary file 1 [file Table_1.DOCX]

**Supplementary Table 1.** Diagnostic and confirmation workflow for target milk pathogens.

| Microorganism / Group | Primary Screening Method | Phenotypic Confirmation | Identification |
| --- | --- | --- | --- |
| *Staphylococcus aureus* | Colony morphology, $\beta$-hemolysis, Gram-positive cocci, Catalase-positive | Coagulase test | Serum agglutination test / MALDI-TOF MS |
| *Streptococcus agalactiae* | Small grey colonies, narrow zone/no hemolysis, Gram-positive cocci, Catalase-negative | CAMP test | Serum agglutination (Lancefield Group B) |
| Other Environmental *Streptococci* (e.g., *S. uberis*, *S. dysgalactiae*) | Esculin hydrolysis, growth on Edward's medium | Biochemical profiling | Serum agglutination test / MALDI-TOF MS |
| *Atypical isolates* | Growth characteristics on selective media | Gram/microscopic staining | MALDI-TOF MS |
| *Prototheca* spp | Growth characteristics on selective media | Lactophenol cotton blue staining | Sanger Sequencing of 16S Region |
